# Next‐generation sequencing of miRNAs in clinical samples of Epstein–Barr virus‐associated B‐cell lymphomas

**DOI:** 10.1002/cam4.1006

**Published:** 2017-02-09

**Authors:** Kouta Sakamoto, Tsuyoshi Sekizuka, Taeko Uehara, Tsunekazu Hishima, Sohtaro Mine, Hitomi Fukumoto, Yuko Sato, Hideki Hasegawa, Makoto Kuroda, Harutaka Katano

**Affiliations:** ^1^ Department of PathologyNational Institute of Infectious Diseases1‐23‐1 ToyamaShinjuku‐ku, Tokyo162‐8640Japan; ^2^ Pathogen Genomics CenterNational Institute of Infectious Diseases1‐23‐1 ToyamaShinjuku‐ku, Tokyo162‐8640Japan; ^3^ Department of PathologyTokyo Metropolitan Komagome Hospital3‐18‐22 HonkomagomeBunkyo‐ku, Tokyo113‐8613Japan

**Keywords:** DLBCL, EBV, lymphoma, miRNA, next‐generation sequencing

## Abstract

Epstein–Barr virus (EBV) encodes 49 microRNAs (miRNAs) in the BART and BHRF1 regions of its genome. Although expression profiles of EBV‐encoded miRNAs have been reported for EBV‐positive cell lines and nasopharyngeal carcinoma, to date there is little information about total miRNA expression, including cellular and viral miRNAs, in the primary tumors of EBV‐associated B‐lymphoproliferative disorders. In this study, next‐generation sequencing and quantitative real‐time reverse transcription‐PCR were used to determine the expression profiles of miRNAs in EBV‐infected cell lines and EBV‐associated B‐cell lymphomas, including AIDS‐related diffuse large B‐cell lymphoma (DLBCL), pyothorax‐associated lymphoma, methotrexate‐associated lymphoproliferative disorder, EBV‐positive DLBCL of the elderly, and Hodgkin lymphoma. Next‐generation sequencing revealed that EBV‐encoded miRNAs accounted for up to 34% of total annotated miRNAs in these cases. Expression of three miR‐BHRF1s was significantly higher in AIDS‐related DLBCL and pyothorax‐associated lymphoma compared with methotrexate‐associated lymphoproliferative disorder and EBV‐positive DLBCL of the elderly, suggesting the association of miR‐BHRF1s expression with latency III EBV infection. Heat map/clustering analysis of expression of all miRNAs, including cellular and EBV miRNAs, by next‐generation sequencing demonstrated that each EBV tumor, except methotrexate‐associated lymphoproliferative disorder, formed an isolated cluster. Principal component analysis based on the EBV‐encoded miRNA expression showed that each EBV tumor formed a distinguished cluster, but AIDS‐related DLBCL and pyothorax‐associated lymphoma formed larger clusters than other tumors. These data suggest that expression of miRNAs, including EBV‐encoded miRNAs, is associated with the tumor type and status of virus infection in these tumors.

## Introduction

Epstein–Barr virus (EBV) is associated with the pathogenesis of malignant tumors, including lymphoma, gastric cancer, and nasopharyngeal carcinoma 1, 2. These EBV‐associated tumors have different patterns of EBV‐encoded mRNA expression, which are categorized as latency types I, II, and III. Type III latency is characterized by the expression of all EBV latent genes, including EBV‐encoded nuclear antigens (EBNAs) and latent membrane proteins (LMPs). In EBV‐associated malignancies with type I and II latency, the expressions of EBV genes are restricted by the activity of a specific promoter. While EBNA1 and EBV‐encoded small RNAs (EBERs) are expressed in almost all types of EBV‐infected cells, the expressions of LMPs and other EBNAs are downregulated in EBV‐positive Burkitt lymphoma and gastric cancer. Different expression profiles are thought to contribute to the pathogenesis of EBV‐associated malignancies 2. LMP1 and EBNA2 are expressed in malignancies of type III latency, such as opportunistic EBV‐associated lymphoma.

MicroRNAs (miRNAs) are short 20–23 nucleotide RNAs 3, 4. EBV encodes 25 pre‐miRNAs and 49 mature miRNAs in two clusters, BHRF1 and BART 5, 6, 7, 8, 9. The BHRF1 cluster encodes three miRNAs, miR‐BHRF1‐1, ‐2, and ‐3, while the BART cluster encodes at least 22 pre‐miRNAs (miR‐BARTs). Both miR‐BHRF1s and miR‐BARTs are expressed in spontaneously EBV‐transformed lymphoblastoid cell lines (LCLs), but expression of miR‐BHRF1s is restricted in EBV‐associated nasopharyngeal carcinoma (NPC) and nasal NK/T‐cell lymphoma 10, 11, 12, 13, 14. In addition, EBV strain B95‐8, a commonly used laboratory strain, has a deletion in the BART cluster, resulting in lack of expression of some miR‐BARTs 15. Since the B95‐8 strain has full transforming potential in human B cells, this indicates that the miR‐BARTs that are missing in B95‐8 are not required for B‐cell transformation 16. However, other studies demonstrated that EBVs that lack the BHRF1 cluster have a reduced ability to transform human B cells, suggesting a contribution of miR‐BHRF1s to B‐cell transformation 17, 18. Furthermore, some EBV‐encoded miRNAs were shown to repress BCL6 expression in diffuse large B‐cell lymphoma 19. Another report demonstrated that miR‐BHRF1‐2 regulated PRDM1/Blimp1, a master regulator of B‐cell terminal differentiation 20. Thus, these reports and other previous studies suggested some contributions by EBV‐encoded miRNAs to the pathogenesis of B‐cell lymphoma 21.

In addition to viral miRNAs, cellular miRNAs also play important roles in the oncogenesis of B‐cell lymphomas 22. miR155 is accumulated in B‐cell lymphoma cells and plays a crucial role in the growth of B‐cell lymphoma 23, 24. miR155 has been shown to suppress activation‐induced cytidine deaminase‐mediated MYC‐IGH translocation in Burkitt lymphoma 25. miR19a and miR19b in Burkitt lymphoma cells are a direct transcriptional target of C‐MYC 26. EBV infection also induces expression of oncomiRs, such as miR‐21 and miR‐146a, in B‐cells 27, 28, 29.

To clarify the roles of cellular and EBV‐encoded mi‐RNAs in vivo, it is important to determine the expression profile of total miRNAs in primary tumors of EBV‐associated lymphoma. To date, the expression profiles of EBV‐encoded miRNAs have been investigated in primary NPC, nasal NK/T‐cell lymphoma, gastric cancer samples, and LCLs using real‐time RT‐PCR 10, 11, 14, 30, 31, 32, 33, 34, 35, 36, 37, but there is little information about EBV‐encoded miRNA expression in primary tumors of EBV‐associated B‐cell lymphoma. Next‐generation sequencing (NGS) is a powerful tool for investigating the expression of small RNAs, including cellular and viral miRNAs. The expression profile of cellular and viral miRNAs has been reported in EBV‐positive diffuse large B‐cell lymphoma (DLBCL) of the elderly and Burkitt lymphoma cases by NGS, DNA microarray, and real‐time PCR analysis 33, 37, 38, 39, 40. However, no report has compared the miRNA expression profiles among different histological categories of EBV‐associated B‐cell lymphomas. In this study, the expression of cellular and EBV‐encoded miRNAs was investigated using NGS and quantitative real‐time RT‐PCR analysis in EBV‐positive cell lines and clinical samples of EBV‐associated B‐cell lymphomas.

## Materials and Methods

### Samples

**Figure 1:**
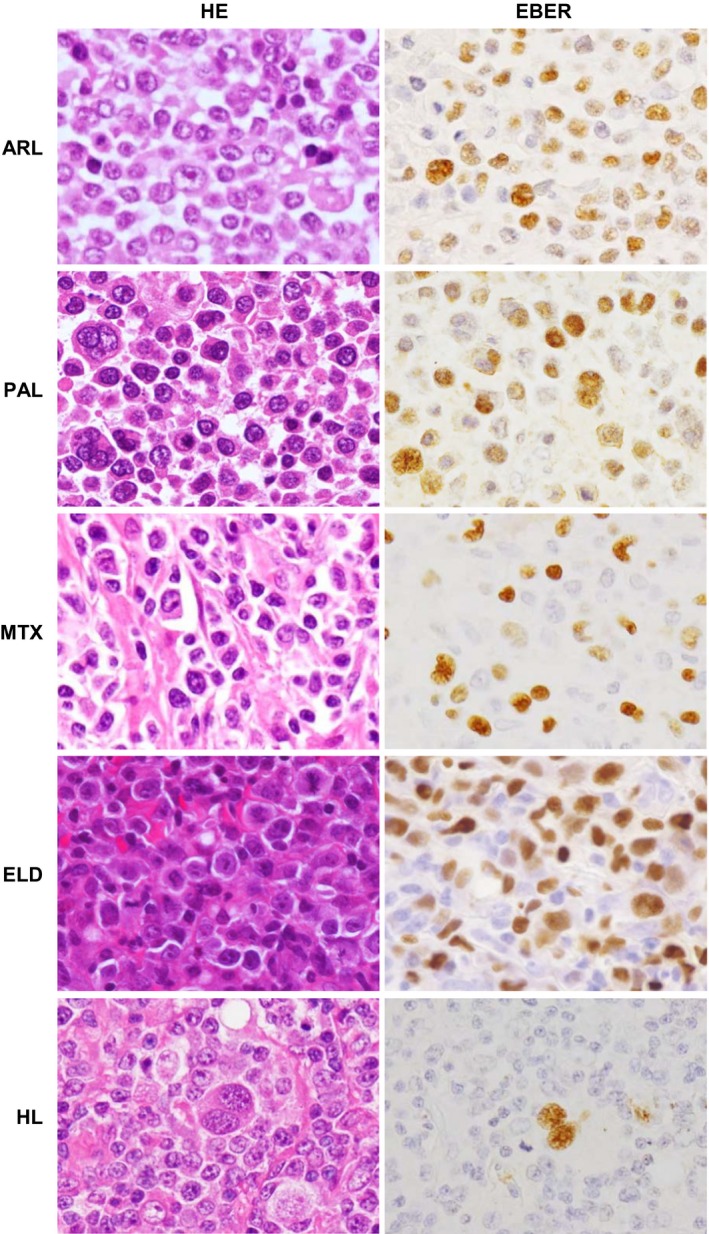
Histological sections of samples used in this study. Hematoxylin‐eosin (HE) stain (left panels) and Epstein–Barr virus (EBV)‐encoded small RNAs (EBERs) in situ hybridization (right panels) are shown in representative cases of EBV‐associated lymphoma. ARL: AIDS‐related diffuse large B‐cell lymphoma, ELD: EBV‐positive diffuse large B‐cell lymphoma (DLBCL) of the elderly, HL: Hodgkin lymphoma, LCL: lymphoblastoid cell line, MTX: methotrexate‐associated lymphoproliferative disorder, PAL: pyothorax‐associated lymphoma.

Studies using human tissue were performed with the approval of the Institutional Review Board of the National Institute of Infectious Diseases (Approval No. 271 and 272) and Tokyo Metropolitan Komagome Hospital (Approval No. 628). All clinical samples were collected from Japanese patients in Japan. All data in this study were analyzed anonymously. Seventeen frozen samples of EBV‐associated diseases were examined by NGS. Samples included three AIDS‐related DLBCL (ARL), four pyothorax‐associated lymphoma (PAL), five methotrexate‐related lymphoma (MTX), three EBV‐positive DLBCL of the elderly (ELD), and two Hodgkin lymphoma (HL) cases. In addition, 18 ARL, four PAL, four MTX, five ELD, and six HL cases were analyzed by real‐time RT‐PCR. The four ARL case samples that were analyzed by real‐time RT‐PCR were formalin‐fixed paraffin‐embedded (FFPE) tissues. Histological diagnosis of lymphoma was based on the 4th edition of World Health Organization classification of lymphoma 41. All cases were confirmed positive for EBV infection using in situ hybridization of EBERs. All EBV‐positive samples contained EBER‐positive cells, but the fraction of positive cells varied among samples (Fig. 1). EBER‐positive cells comprised 30–100% of the cells in ARL, PAL, MTX, and ELD samples. In the HL samples, only the Reed–Sternberg cells were positive for EBER, but they were less than 10% of all cells. All ARL and PAL cases were positive for LMP1 and EBNA2 by immunohistochemistry or RT‐PCR, suggesting latency III. Cases of MTX and ELD were negative for LMP1, but some of them were not examined. HL cases were positive for LMP1 and negative for EBNA2 in Reed–Sternberg cells, suggesting latency II. Histological subtypes of DLBCL included centroblastic and immunoblastic variants, but some cases were not distinguishable histologically. Six EBV‐negative DLBCL tumors from immunocompetent patients were also examined. In addition to the tumors from clinical cases, one EBV‐transformed EBV LCL (spontaneous transformation), four EBV (strain B95‐8)‐transformed LCLs, and two EBV‐negative and KSHV‐positive primary effusion lymphoma cell lines, TY‐1 and BCBL‐1, were examined.

### RNA extraction

Total RNA, including miRNA, was extracted from frozen or paraffin‐embedded samples and cell lines using the High Pure miRNA purification kit (Roche Molecular Biochemicals, Indianapolis, IN) and subsequently treated with Turbo DNase (Ambion, Austin, TX), all according to instructions from the manufacturers.

### NGS

Small RNA libraries were established with the TruSeq small RNA kit (Illumina, San Diego, CA) from 18‐ to 35‐nucleotide cDNAs using 5 *μ*g of DNase‐treated total RNA. Small RNA was sequenced using the Illumina Genome Analyzer IIx platform with cBot‐SR v2 and SBS 36 cycle v5 kit. Sequence reads were analyzed with CLC Genomics Workbench (version 7.5.1, CLC bio, Aarhus, Demark). miRBase release 21 was used as the miRNA database (http://www.mirbase.org/). Homo_sapiens.GRCh37.57.ncrna was used as a comprehensive noncoding RNA database (http://www.ncrna.org/). All annotated reads matching to pre‐miRNA were counted as miRNA reads.

### Real‐time RT‐PCR for miRNA

Total RNA (100 ng) was reverse‐transcribed using the miScript Reverse Transcription Kit from Qiagen (Valencia, CA). Real‐time reverse transcription polymerase chain reaction (RT‐PCR) for the quantification of 42 EBV‐encoded miRNAs and human cellular miRNAs miR16 and miR21 was carried out with the miScript PCR system (Qiagen). Ratios of the copy numbers of EBV‐encoded miRNA to miR16 were calculated as follows: ratio of target miRNA to miR16 = 2^Ct of miR16^/2^Ct of target^ (Ct = cycle threshold). Each Ct was determined by results of EBV‐negative controls for EBV‐encoded miRNA and nontemplate controls for miR16 in each experiment. In addition, stem‐loop real‐time RT‐PCR (Taqman microRNA assay, Applied Biosystems, Foster City, CA) was also performed for comparison with the miScript PCR system. Synthesized miR21 with deletion or addition of single or double nucleotides in the 3′ end (Fasmac, Tokyo, Japan) was examined.

### Heat map and cluster analysis

Heat map images were produced using TreeView and Cluster software by Michael Eisen, University of California at Berkeley (http://rana.lbl.gov/EisenSoftware.htm) 42.

### Principal component analysis

Principal component analysis (PCA) was performed on normalized data of ratios of EBV‐encoded miRNA copy number to miR16 copy number using the PCA function of SPSS software (IBM, Armonk, NY). The first three principal components were used to produce two‐dimensional and three‐dimensional plots. Resolution and convex hulls in three‐dimensional plots were calculated and plotted using MATLAB (MathWorks, Natick, MA), as reported previously 31. Volumes of convex hulls were measured by MATLAB ConvexHull function after Delaunay‐triangulation.

### Accession number

The annotated miRNAs detected in cell lines and clinical samples by NGS in this study are registered as accession number DRA002823 in the DDBJ Sequence Read Archive.

### Statistical analysis

Mann–Whitney U‐test was used for nonparametric two‐group comparison (Graph Pad Prism 5, GraphPad Software, La Jolla, CA).

## Results

### Small RNA libraries

Small RNA libraries were established from an EBV‐transformed LCL and 17 clinical samples of EBV‐associated lymphoproliferative disorders to determine the miRNA profiles. Although more than 100,000 reads were sequenced in each sample by NGS, unannotated rates ranged from 13.91% to 65.61% (Table S1). Noncoding RNA reads ranged from 14.43% to 79.38% and miRNA reads ranged from 1.16% to 71.66%. A total of 688 cellular and EBV miRNAs were annotated from all small RNA libraries (Table S2). EBV‐encoded miRNAs ranged from 0.03% to 34.7% of all annotated miRNA reads, and their percentages varied among cases (Table S1).

### Expression profile of miRNAs in EBV‐infected clinical samples using NGS

**Figure 2:**
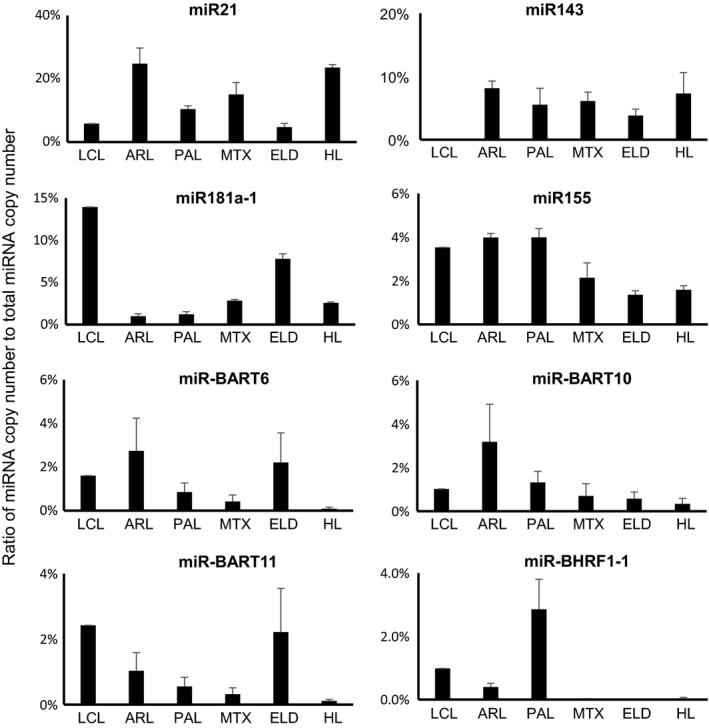
Cellular and Epstein–Barr virus (EBV)‐encoded miRNA expression in LCL and clinical samples. Ratio of miRNA copy number to total miRNA copy number is shown on the vertical axis. Four frequent cellular and viral miRNAs are shown. Error bars indicate standard error.

**Figure 3:**
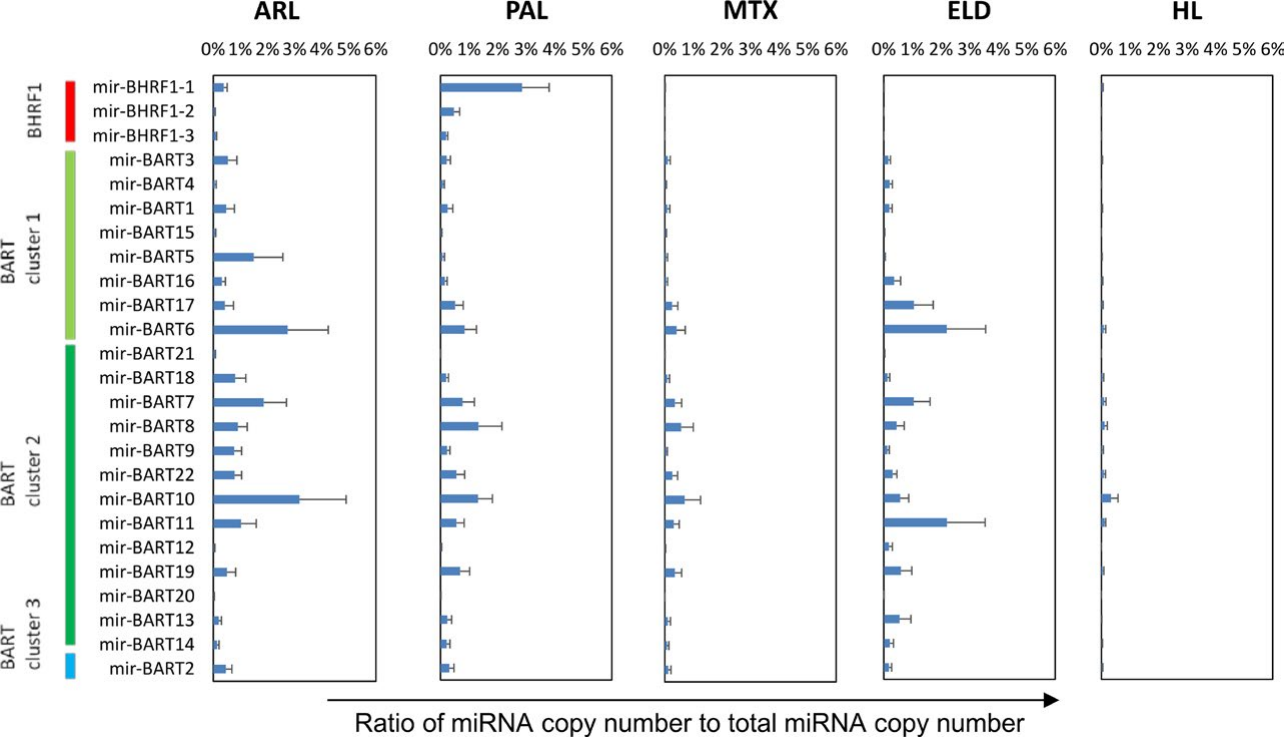
Epstein–Barr virus (EBV)‐encoded miRNA expressions by NGS **.** Ratios of EBV miRNA reads to total miRNA reads are indicated by percentage in the horizontal axis. Error bars indicate standard error.

Among all annotated miRNAs, the most abundant miRNA in the average expression of all samples was miR21, a cellular miRNA, followed by miR143, ‐181a, ‐10b, ‐92a, ‐7641‐2, ‐22, and ‐155 (Table 1). About 25% of all annotated miRNAs was miR21 in ARL and HL samples (Fig. 2). miR143, which is associated with angiogenesis 43, 44, was detected in 3.8% to 8.1% of total miRNA reads in EBV‐associated lymphoma cases, but in only 0.01% in LCL (Fig. 2). miR155, which is associated with B‐cell lymphomagenesis 45, was also expressed constantly among EBV‐associated lymphoma cases. Among the EBV‐encoded miRNAs, miR‐BART6, ‐10 and ‐11 and miR‐BHRF1‐1 were frequently detected in the cases (Table 1 and Figs. 2 and 3). miR‐BHRF1‐1 was expressed higher in the group of LCL, ARL, and PAL than in the group of MTX, ELD, and HL (Figs. 2 and 3, Mann–Whitney U‐test, *P *<* *0.01).

**Table 1:** Detection rates of miRNAs in each category

miRNA	LCL	ARL	PAL	MTX	ELD	HL	Average	S.D.
miR21	5.78%	24.79%	10.39%	15.07%	4.67%	23.57%	14.05%	8.68%
miR143	0.01%	8.14%	5.54%	6.11%	3.82%	7.30%	5.15%	2.93%
miR181a‐1	13.96%	0.97%	1.20%	2.84%	7.79%	2.57%	4.89%	5.08%
miR181a‐2	14.15%	0.94%	1.19%	2.77%	7.73%	2.51%	4.88%	5.16%
miR10b	0.02%	4.83%	5.86%	4.74%	0.90%	1.33%	2.95%	2.47%
miR92a‐1	2.75%	0.79%	2.64%	1.87%	6.01%	0.80%	2.48%	1.93%
miR7641‐2	0.02%	0.12%	6.12%	4.80%	0.32%	5.34%	2.79%	2.92%
miR92a‐2	2.48%	0.70%	2.38%	1.62%	5.24%	0.70%	2.19%	1.68%
miR22	0.74%	2.21%	2.83%	4.85%	1.96%	1.93%	2.42%	1.37%
miR155	3.52%	3.98%	3.99%	2.13%	1.36%	1.59%	2.76%	1.21%
miR191	10.21%	0.62%	2.65%	1.61%	2.24%	1.43%	3.13%	3.54%
miR142	1.36%	2.35%	1.30%	2.78%	0.55%	6.16%	2.42%	2.00%
let‐7i	0.44%	1.77%	1.73%	2.46%	3.46%	1.89%	1.96%	0.99%
miR146b	2.23%	1.57%	1.73%	1.57%	1.15%	3.21%	1.91%	0.73%
miR146a	2.98%	4.06%	0.39%	0.66%	0.35%	2.87%	1.89%	1.61%
miR26a‐2	0.72%	1.37%	1.23%	2.14%	1.08%	1.47%	1.34%	0.47%
miR26a‐1	0.72%	1.36%	1.21%	2.15%	1.07%	1.47%	1.33%	0.48%
miR27b	0.72%	0.57%	1.30%	1.74%	1.66%	0.91%	1.15%	0.49%
miR10a	0.01%	1.78%	2.45%	0.82%	0.32%	1.02%	1.07%	0.91%
miR‐BART6	1.60%	2.74%	0.85%	0.42%	2.21%	0.09%	1.32%	1.04%
miR‐BART10	1.02%	3.18%	1.32%	0.70%	0.58%	0.34%	1.19%	1.03%
miR7641‐1	0.01%	0.02%	2.06%	1.51%	0.14%	1.66%	0.90%	0.94%
miR30e	1.13%	0.62%	1.19%	1.23%	0.36%	1.33%	0.98%	0.39%
miR378a	1.68%	0.73%	0.98%	0.56%	1.07%	0.45%	0.91%	0.44%
miR4454	0.01%	0.02%	0.71%	1.10%	0.39%	4.41%	1.11%	1.67%
miR‐BART11	2.42%	1.03%	0.56%	0.32%	2.21%	0.11%	1.11%	0.99%
miR126	0.00%	0.43%	1.10%	1.49%	0.26%	1.12%	0.73%	0.58%
let‐7f‐2	1.40%	0.61%	0.65%	1.03%	0.61%	0.72%	0.84%	0.32%
let‐7f‐1	1.35%	0.60%	0.62%	0.99%	0.60%	0.71%	0.81%	0.30%
miR‐BART8	2.36%	0.91%	1.34%	0.58%	0.45%	0.11%	0.96%	0.80%
mir‐BART7	1.11%	1.86%	0.77%	0.35%	1.05%	0.09%	0.87%	0.63%
mir‐BHRF1‐1	0.99%	0.39%	2.85%	0.01%	0.01%	0.03%	0.71%	1.11%

S.D.: standard deviation; EBV, Epstein–Barr virus; HL, Hodgkin lymphoma.

The 32 most frequently detected miRNAs are listed. Percentages of the miRNA in total annotated miRNAs are shown. EBV‐encoded miRNAs are underlined. LCL was a spontaneously EBV‐transformed lymphoblastoid cell line. Averages of all samples including both cell lines and clinical samples are shown in the right.

**Figure 4:**
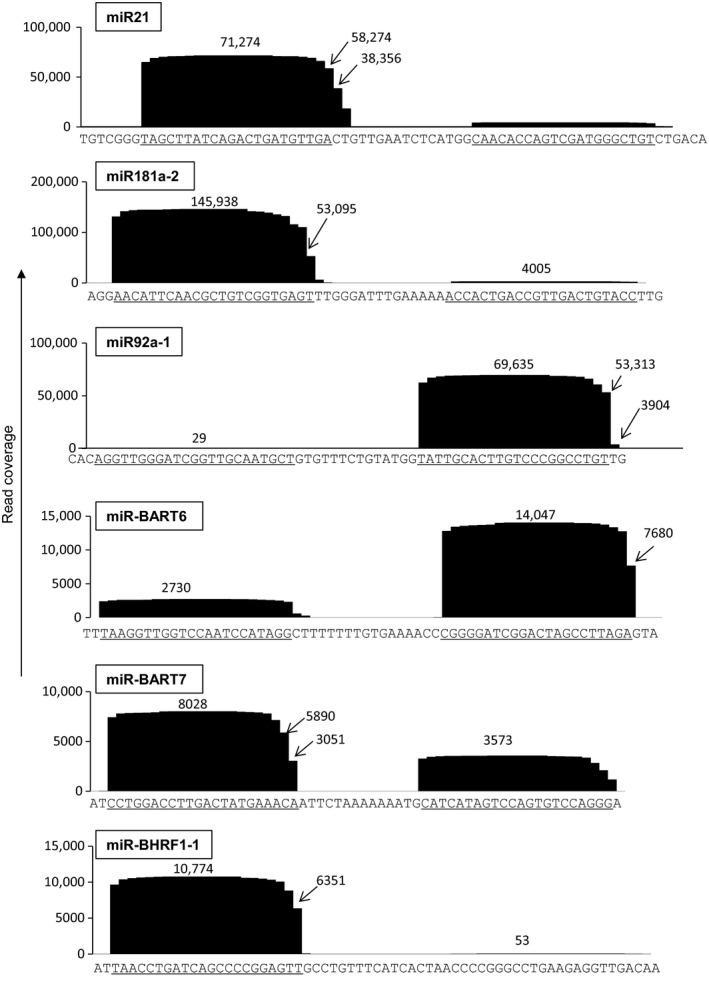
Coverage of reads in each pre‐miRNA in spontaneously transformed LCL samples. Three frequent cellular and EBV miRNAs are shown. Sequences of pre‐miRNAs are shown under the figures. Under bars indicate 5p and 3p miRNAs. Vertical axis indicates coverage (reads). EBV, Epstein–Barr virus.

Since all reads matching to pre‐miRNAs were counted as miRNAs in this system, the read counts contained both 5p and 3p mature miRNAs but also immature miRNAs. We also investigated the profile of coverage in each pre‐miRNA. Coverage profiles in pre‐miRNAs demonstrated frequent deletion or addition of a single nucleotide in the 3′ end of mature miRNAs in both cellular and EBV‐encoded miRNAs in a spontaneous transformed LCL (Fig. 4). Among 20 miRNAs picked up randomly from cellular and viral miRNAs, the frequencies of changes in the 3′ end of mature miRNAs were not different between those of cellular and viral miRNAs (data not shown). In addition, frequencies of changes in the 3′ end of mature miRNAs were not different between miRNAs frequently expressed in clinical samples of EBV‐associated lymphomas and those in LCL (data not shown).

### Expression profile of EBV‐encoded miRNAs using real‐time RT‐PCR

**Figure 5:**
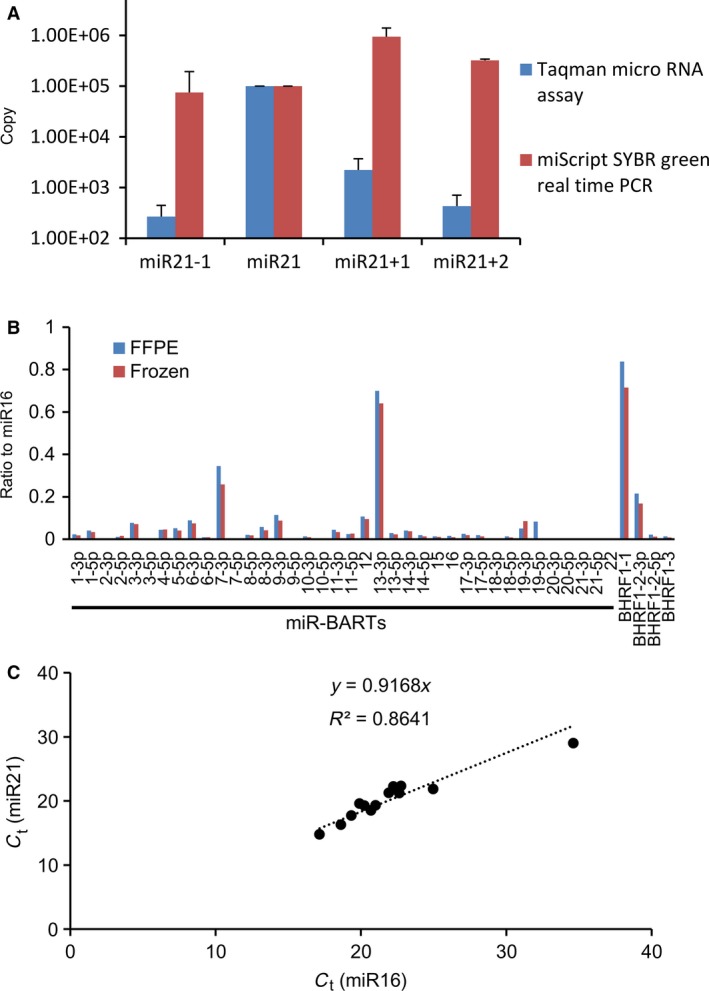
Conditions of real‐time PCR. (A) Detection of mature miRNA with deletion or addition in the 3′ end by real‐time RT‐PCR. A total of 1 × 10^5^ copies of synthesized miR21, miR21 with deletion (miR21‐1), miR21 with addition of single nucleotide (miR21 + 1), and miR21 with addition of two nucleotides (miR21 + 2) in the 3′ end of mature miRNA were examined with the miScript PCR system (miScript SYBR green real‐time PCR) and stem‐loop real‐time RT‐PCR (Taqman microRNA assay). Error bars indicate standard error for triplicate analyses. (B) Comparison of formalin‐fixed paraffin‐embedded (FFPE) and frozen samples by real‐time RT‐PCR. miR‐BARTs and miR‐BHRF1s were quantified by the miScript PCR system. FFPE and frozen samples were obtained from xenotropically inoculated lymphoma tissues in severe combined immunodeficiency mice. (C) Cycle thresholds (Ct) for miR16 and miR21. The copy numbers of two cellular miRNAs, miR16 and miR21, were measured and plotted in 12 representative clinical samples using the miScript PCR system. Linear approximation line (broken line) and correlation coefficient (R^2^) are indicated.

**Figure 6:**
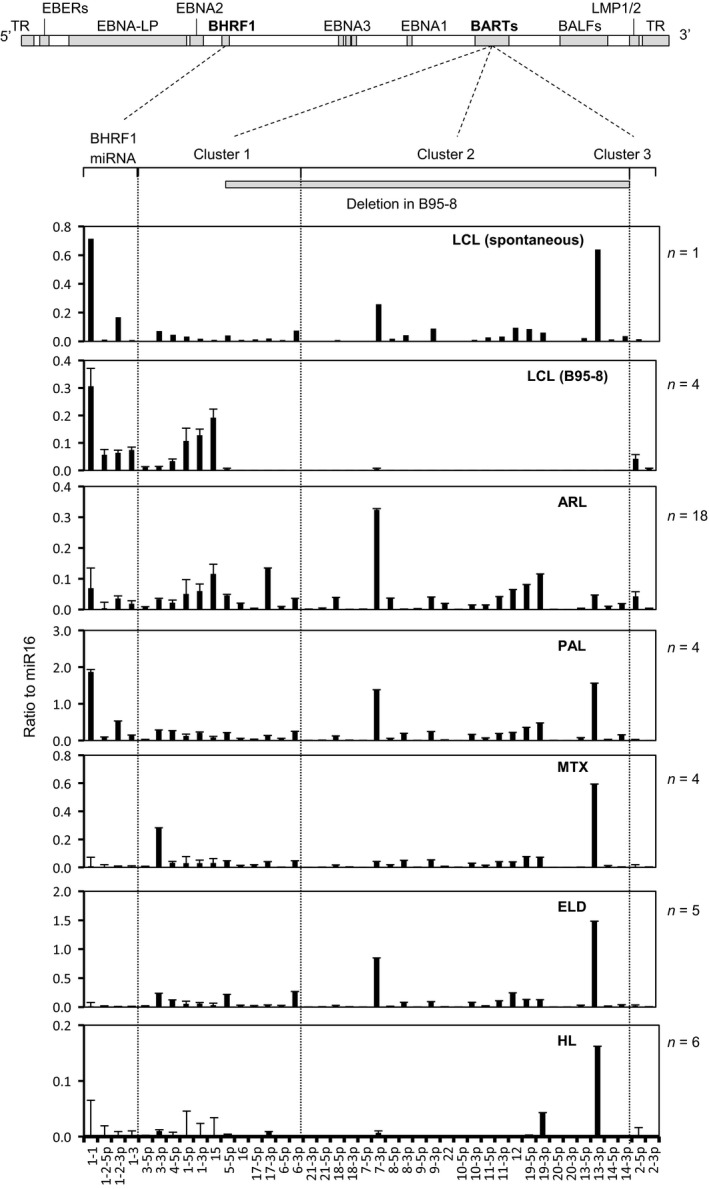
Epstein–Barr virus (EBV)‐encoded miRNA expression in LCL and clinical samples by real‐time RT‐PCR. The EBV genome map is shown at the top. Ratios of copy numbers of EBV‐encoded miRNA to miR16 are shown in each category. A number of samples are shown in the right of each panel. Error bars indicate standard error. Deletion in the middle portion of the BART gene in B95‐8 is indicated by a gray bar (139,724‐151,554 nt in GenBank NC_007605). EBV‐encoded miRNAs were not detected in the EBV‐negative cell lines, TY‐1 and BCBL‐1, and 6 EBV‐negative cases of diffuse large B‐cell lymphoma (DLBCL) (data not shown).

To confirm the expression profile of EBV‐encoded mi‐RNAs, we performed real‐time RT‐PCR of these EBV‐encoded miRNAs using additional cases of EBV‐associated lymphoma (18 ARL, 4 PAL, 4 MTX, 5 ELD, and 6 HL cases). Since NGS demonstrated frequent deletion or addition of a single nucleotide in the 3′ end of mature miRNA, we first compared the miScript PCR system and stem‐loop real‐time RT‐PCR by examining synthesized miR21 that contained a deletion or addition of one or two nucleotides in the 3′ end. The miScript PCR system succeeded in detecting miR21 with deletions and additions, whereas stem‐loop real‐time PCR detected miR21 with deletion or addition with less than one‐tenth sensitivity (Fig. 5A). Thus, the miScript PCR system was employed to detect 5p and 3p mature miRNAs separately. Real‐time RT‐PCR analysis revealed a similar profile of EBV miRNAs between FFPE tissues and frozen samples of xenotropically inoculated lymphoma tissues in severe combined immunodeficiency mice (Fig. 5B). In addition, cycle thresholds of miR16 were proportional to those of miR21 in 12 representative clinical samples (Fig. 5C); therefore, the copy numbers of EBV‐encoded miRNAs can be normalized by those of miR16 in each sample. Real‐time RT‐PCR revealed that the pattern of EBV‐encoded miRNA expression in ARL is similar to PAL (Fig. 6). miR‐BHRF1‐1 showed higher copy numbers in ARL and PAL than in MTX, ELD, and HL (Mann–Whitney U‐test, *P *<* *0.01). In HL, we detected miR‐BARTs 13‐3p and 19‐3p, with no or low level expression of the other miRNAs. In addition, total expressions of EBV‐encoded miRNAs were low in MTX and HL cases compared with other cases (Mann–Whitney U‐test, *P *<* *0.01).

### Heat map, cluster analysis, and PCA

**Figure 7:**
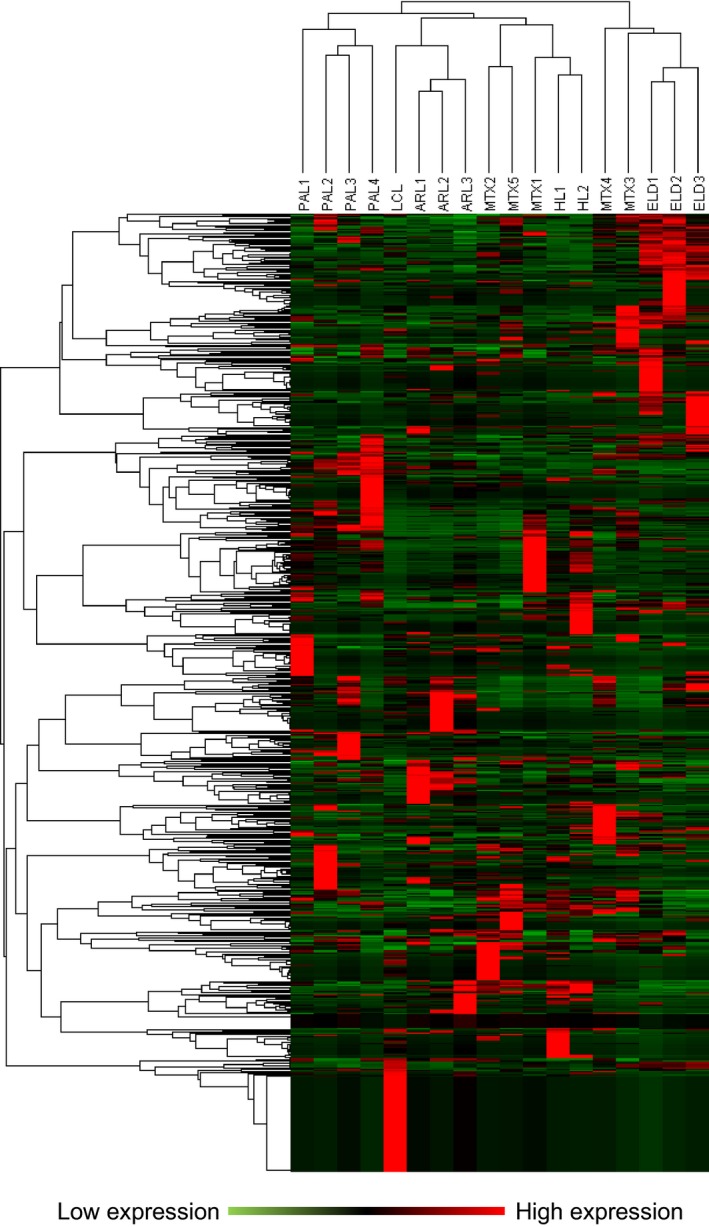
Heat map and cluster analysis. Heat map and cluster analysis of miRNA expression using data of NGS. Individual patient samples are shown in columns and miRNAs including both cellular and EBV miRNAs in rows. Expression measured by NGS is displayed in red color and green color depending on expression above or below median expression level.

**Figure 8:**
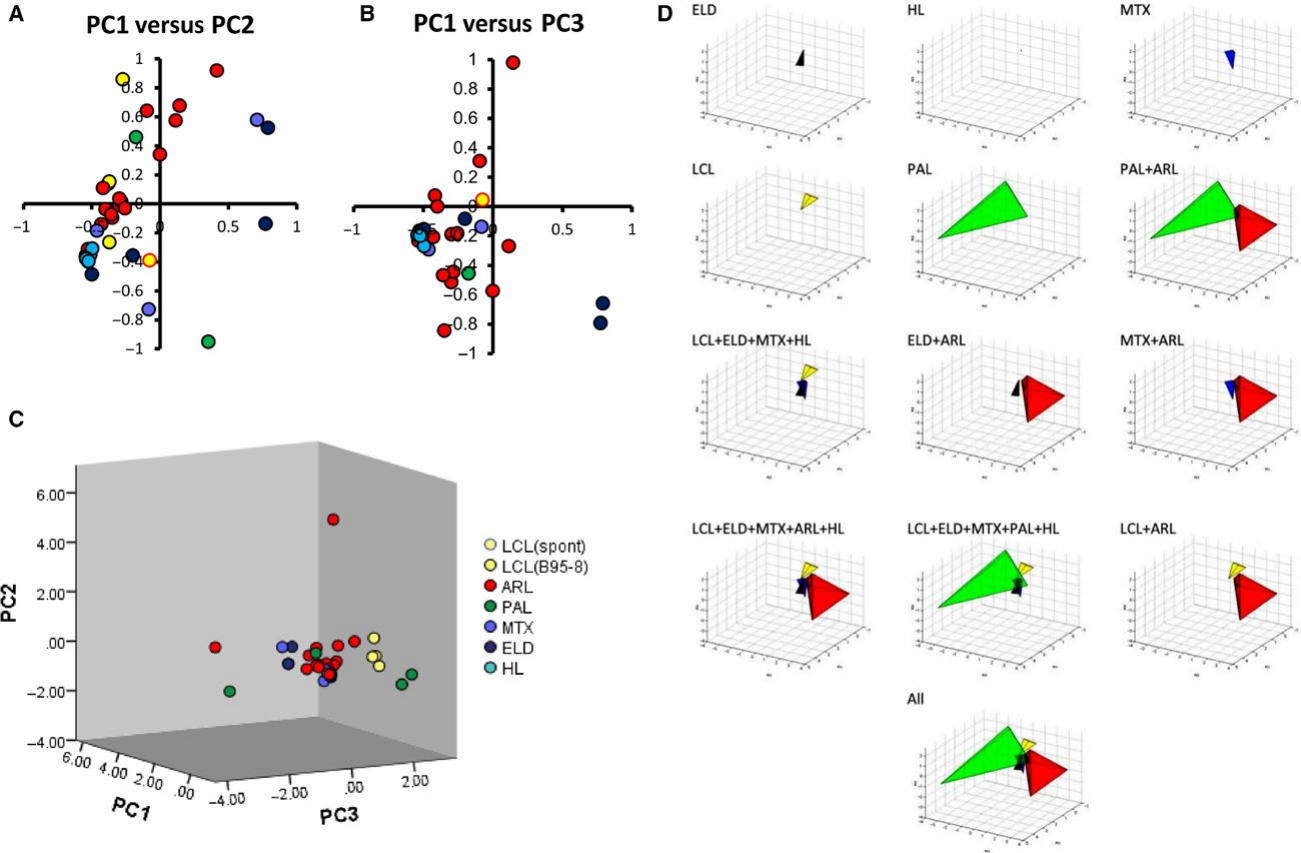
Principal component analysis (PCA) on the data of Epstein–Barr virus (EBV) miRNA expression by real‐time PCR. PCA was based on the EBV‐encoded miRNA expression data analyzed by real‐time RT‐PCR. The sample size of each category was same as in Figure 6. (A‐B) Two‐dimensional plots of samples using first and second (A), and first and third (B) principal components (PC). (C) Three‐dimensional plots of samples using the first three components. (D) PCA three‐dimensional plots of grouped samples using the first three components. Clusters of samples in the groups are shown by different colors in three‐dimensional plots of PCA using PC1, PC2, and PC3.

To determine if EBV‐associated lymphoma cases were categorized based on expression patterns of miRNAs, we performed cluster analysis on our data set using heat maps and PCA. Heat map and cluster analysis based on the data by NGS demonstrated that ARL, PAL, HL, and ELD formed an individual cluster based on the data of total miRNA expression, including both cellular and viral miRNAs (Fig. 7). MTX cases belonged to various clusters and did not form a cluster. PCA based on the data by EBV‐encoded miRNA expression by real‐time RT‐PCR analysis showed small volumes of cluster in three‐dimensional plots of PC1‐3 corresponding to LCL (cluster volume = 0.023), HL (1.62 × 10^−5^), MTX (8.63 × 10^−4^), and ELD (0.029) samples, whereas ARL (1.426) and PAL (2.030) formed larger clusters (Fig. 8). PCA showed that each EBV tumor, other than ARL and PAL, formed a distinguished cluster, but samples of ARL and PAL distributed broadly in the three‐dimensional plots of PC1‐3 (Fig. 8D).

## Discussion

In this study, we describe the expression profiles of cellular and viral miRNAs in primary tumors of EBV‐associated lymphoma. High expression of miR21 is common among many types of tumors, including B‐cell lymphoma 46, 47, 48. In addition, miR21 was identified as a useful marker for primary diffuse large B‐cell lymphoma of the central nervous system in the cerebrospinal fluid 49. miR143 has a possible role in angiogenesis in tumors, suggesting an association between miR143 expression and lymphoma growth 43. Downregulation of miR143 was demonstrated in B‐cell lymphoma cell lines 50. In this study, NGS revealed 0.008% of total annotated reads of miRNA in an EBV‐transformed LCL (Fig. 2). Low expression of miR143 in EBV‐positive cell lines was previously reported, such as 0.003% in Jijoye cells, 0.0004% in LCL35, 0.0002% in LCL‐BAC, and 0.0007% in SDLCL in total annotated reads of miRNAs 7, 51. On the other hand, this study demonstrated that miR‐143 comprised 6.07% of total miRNAs on average in clinical samples. This observation indicates a difference in miRNA expression between cell lines and clinical primary cases. We also confirmed high copy number of miR155 in EBV lymphoma in this study. Lower copy number of miR155 in ELD and HL than ARL and PAL may be associated with the presence of nontumor cells and inflammatory cells in the samples (Fig. 2). In particular, tumor cells are observed at low frequency in the lymph node with HL, whereas almost all cells were tumor cells in tumor tissues of ARL and PAL (Fig. 1).

Several reports have described expression levels of EBV‐encoded miRNAs using stem‐loop RT‐PCR 11, 52, 53 and NGS 33 in EBV‐associated diseases. In our study, NGS identified frequent variants of mature miRNAs with 3′ deletion and addition (Fig. 4), which the stem‐loop RT‐PCR was unable to detect (Fig. 5). This is important information for detecting miRNAs using the stem‐loop primer, because the stem‐loop RT‐PCR may fail to detect large portion of miRNAs with such a deletion or addition of nucleotide in the 3′ region.

In this study, the miScript PCR system failed to detect almost all miRNAs encoded by BART cluster 2 in the LCL transformed by B95‐8 EBV (Fig. 6). These results were reasonable, as cluster 2 is missing in B95‐8, which has been widely used in the establishment of LCLs. miR‐BARTs in cluster 2 were not detected in LCLs transformed by B95‐8 EBV, but were detected at high levels in spontaneously transformed LCLs, ARL, and other clinical samples. A recent study revealed that at least 8 miRNAs in BART cluster 2 (miR‐BARTs 15, 18‐3p, 7‐5p, 10‐3p, 10‐5p, 11‐3p, 13‐5p, and 14‐5p) were identified as associated with latency type III infection of EBV 31. Our data confirmed this, in part. For example, miR‐BARTs 15, 10‐3p, 11‐3p, and 14‐3p were expressed at higher levels in ARL than other tumors. However, expression levels of miR‐BARTs 7‐5p, 18‐5p, and 13‐5p were low in all samples. These observations are consistent with previous studies investigating lymphoma samples 31, 33, although others reported no impact of miR‐BARTs on LCL growth or survival in vitro 15. As reported previously 7, 51, 54, miR‐BHRF1 expression is associated with latency III infection of EBV. Our results by NGS and real‐time RT‐PCR demonstrate relatively high copies of miR‐BHRF1‐1, ‐2, and ‐3 expressions in ARL and PAL, both of which are latency III infection of EBV. Thus, our data suggest that miRNA expression differs between tumors and may be associated with EBV latency, in part.

PCA showed a broad distribution of ARL and PAL samples, but close clusters were formed corresponding to HL, MTX, ELD in two‐ and three‐dimensional plots (Fig. 8), suggesting that EBV‐encoded miRNA expression was deregulated in ARL and PAL. One study reported that EBV‐encoded miRNAs were strictly regulated in normal EBV‐positive nontumor B‐cells, and that deregulation of EBV‐encoded miRNA expression was observed only in tumor cells 31. The results of heat map and PCA in this study suggest that EBV‐encoded miRNA expression was partially regulated in HL, MTX, and ELD but drastically deregulated in ARL and PAL. The most probable difference in clinical manifestations between ARL/PAL and others is the immune status of the host. While the mechanism of viral miRNA expression is unknown, the immune status of the host may affect viral miRNA expression. Further studies are required to reveal details of the association between viral miRNA expression and host immunity.

## Conflict of interests

The authors have declared that no competing interests exist.

## Supporting information


**Table S1**. Overview of small RNAs detected by NGS.

**Table S2**. Numbers of annotated miRNA reads in each small RNA library.
