# Supplementary material for: Next‐generation sequencing of miRNAs in clinical samples of Epstein–Barr virus‐associated B‐cell lymphomas
Source: Cancer Med. 2017 Feb 9;6(3):605–18. doi: 10.1002/cam4.1006 (PMC5345668; doi:10.1002/cam4.1006)
Supplement: Supplementary file 1 — Table S1. Overview of small RNAs detected by NGS. [file CAM4-6-605-s001.docx]

**Supplementary Table S1. Overview of small RNAs detected by a NGS.** ARL: AIDS-related diffuse large B-cell lymphoma, EBV: Epstein-Barr virus, ELD: EBV-positive DLBCL of the elderly, HL: Hodgkin lymphoma, LCL: lymphoblastoid cell line, MTX: methotrexate-associated lymphoproliferative disorder, PAL: pyothorax-associated lymphoma.

|  |  |  | **miRNA reads** | | | | | | **human non-coding RNA** | | **Unannotated reads** | |
| --- | --- | --- | --- | --- | --- | --- | --- | --- | --- | --- | --- | --- |
| **Category** | **Sample** | **Total reads** | **miRNA reads** | **% of total reads** | **Human miRNA** | **Human miRNA in all miRNA reads (%)** | **EBV miRNA** | **EBV miRNA in all miRNA reads (%)** | **Reads** | **% of total reads** | **Reads** | **% of total reads** |
| **Cell line** | **LCL** | **1,659,342** | **1,189,071** | 71.66% | 1,005,839 | 84.59% | 183,232 | 15.41% | **239,486** | 14.43% | **230,785** | 13.91% |
| **ARL (n=3)** | **ARL1** | **1,024,844** | **48,909** | 4.77% | 45,654 | 93.34% | 3,255 | 6.66% | **813,514** | 79.38% | **162,421** | 15.85% |
|  | **ARL2** | **1,035,005** | **160,713** | 15.53% | 105,005 | 65.34% | 55,708 | 34.66% | **655,165** | 63.30% | **219,127** | 21.17% |
|  | **ARL3** | **2,149,366** | **136,164** | 6.34% | 121,369 | 89.13% | 14,795 | 10.87% | **1,327,212** | 61.75% | **685,990** | 31.92% |
| **PAL (n=4)** | **PAL1** | **1,806,366** | **79,738** | 4.41% | 70,888 | 88.90% | 8,850 | 11.10% | **700,783** | 38.80% | **1,025,845** | 56.79% |
|  | **PAL2** | **552,620** | **41,936** | 7.59% | 39,338 | 93.80% | 2,598 | 6.20% | **219,579** | 39.73% | **291,105** | 52.68% |
|  | **PAL3** | **243,755** | **12,672** | 5.20% | 9,580 | 75.60% | 3,092 | 24.40% | **116,860** | 47.94% | **114,223** | 46.86% |
|  | **PAL4** | **727,535** | **16,026** | 2.20% | 14,597 | 91.08% | 1,429 | 8.92% | **354,064** | 48.67% | **357,445** | 49.13% |
| **MTX (n=5)** | **MTX1** | **978,283** | **11,359** | 1.16% | 11,251 | 99.05% | 108 | 0.95% | **332,792** | 34.02% | **634,132** | 64.82% |
|  | **MTX2** | **1,944,281** | **399,628** | 20.55% | 391,702 | 98.02% | 7,926 | 1.98% | **572,398** | 29.44% | **972,255** | 50.01% |
|  | **MTX3** | **742,327** | **52,899** | 7.13% | 52,101 | 98.49% | 798 | 1.51% | **271,324** | 36.55% | **418,104** | 56.32% |
|  | **MTX4** | **417,663** | **48,952** | 11.72% | 41,014 | 83.78% | 7,938 | 16.22% | **165,885** | 39.72% | **202,826** | 48.56% |
|  | **MTX5** | **591,772** | **210,374** | 35.55% | 209,838 | 99.75% | 536 | 0.25% | **177,902** | 30.06% | **203,496** | 34.39% |
| **ELD (n=3)** | **ELD1** | **833,165** | **113,734** | 13.65% | 113,704 | 99.97% | 30 | 0.03% | **201,810** | 24.22% | **517,621** | 62.13% |
|  | **ELD2** | **469,635** | **52,619** | 11.20% | 47,071 | 89.46% | 5,548 | 10.54% | **118,639** | 25.26% | **298,377** | 63.53% |
|  | **ELD3** | **421,763** | **56,761** | 13.46% | 44,247 | 77.95% | 12,514 | 22.05% | **88,277** | 20.93% | **276,725** | 65.61% |
| **HL (n=2)** | **HL1** | **1,983,584** | **178,396** | 8.99% | 174,701 | 97.93% | 3,695 | 2.07% | **792,745** | 39.97% | **1,012,443** | 51.04% |
|  | **HL2** | **2,840,538** | **100,148** | 3.53% | 99,777 | 99.63% | 371 | 0.37% | **1,298,757** | 45.72% | **1,441,633** | 50.75% |
